# P05 The role of JAK inhibitors in difficult-to-treat adult-onset Still’s disease: a case series

**DOI:** 10.1093/rap/rkae117.036

**Published:** 2024-11-05

**Authors:** Eman Elfar, Arti Mahto

**Affiliations:** King’s College Hospital, London, United Kingdom; King’s College Hospital, London, United Kingdom

## Abstract

**Introduction:**

Adult-onset Still’s Disease (AOSD) is a rare systemic auto-inflammatory disorder characterised by fever, arthritis and rash. Some patients with AOSD exhibit resistance or intolerance to conventional treatments, necessitating alternative therapeutic strategies.

This case series aims to explore the efficacy and safety of JAK inhibitors in managing difficult-to-treat AOSD. The driving force behind Still’s disease lies in the excessive and inappropriate production of pro-inflammatory cytokines, notably IL-6 and interferon. Given the pivotal role of JAK inhibitors in mitigating these cytokine pathways, this study highlights their potential therapeutic impact in the management of difficult to treat AOSD.

**Case description:**

In this retrospective case series, three adult patients with difficult-to-treat AOSD were evaluated for their response to JAK inhibitors, specifically tofacitinib and baricitinib.

**Case 1:** A 32-year-old male presented with a three-week history of fevers, night sweats, weight loss, and lymphadenopathy. Diagnosed with AOSD 18 months later, his treatment began with prednisolone and methotrexate, which was subsequently switched to azathioprine. Tocilizumab was then introduced, alleviating joint pain but causing dermatitis. He was switched to anakinra, allowing a reduction in prednisolone but failing to induce remission and resulting in abnormal liver functions and increased hepatitis B virus titers. Finally, he commenced tofacitinib, achieving symptom resolution, reduced serum ferritin levels, and cessation of steroids.

**Case 2:** A 32-year-old woman presented with a six-month history of joint pain, swelling, stiffness, night sweats, weight loss, and a recurrent rash. Laboratory tests showed anemia, lymphopenia, elevated inflammatory markers, and high ferritin levels. Initial treatment with 60 mg oral prednisolone showed some response, but polyarthritis necessitated further intervention. Anakinra initially managed systemic symptoms, though joint swelling persisted. Tocilizumab was tried but proved ineffective. Subsequently, transitioning to baricitinib at 4 mg daily resulted in good symptomatic control.

**Case 3:** An 18-year-old female presented with recurrent fevers, an intermittent macular rash, joint pains, diarrhoea and splenomegaly. Despite initial antibiotic treatment, persistent symptoms led to an AOSD diagnosis, prompting steroid therapy. Methotrexate failed to control symptoms and caused significant nausea, leading to a switch to tocilizumab. While anakinra initially resolved skin and joint symptoms, recurrent flares necessitated further changes. Azathioprine was trialed but discontinued due to side effects. Colchicine was tried next. Finally, tofacitinib was introduced, and one year later, she achieved significant progress, reducing prednisolone dosage to 5 mg.

**Discussion:**

The findings from this case series underscore the potential of JAK inhibitors, particularly tofacitinib and baricitinib, as effective treatments for refractory AOSD. In all three cases, patients showed substantial clinical improvement and were able to reduce or discontinue steroids. This suggests a promising role for JAK inhibitors in managing AOSD, especially given the disease’s association with excessive pro-inflammatory cytokine production, including IL-6 and IFN pathways.

The transition to JAK inhibitors was often necessitated by inadequate responses or adverse effects from conventional therapies like methotrexate, azathioprine, and biologics such as tocilizumab and anakinra. The successful outcomes with JAK inhibitors highlight their ability to achieve disease control where other treatments failed, demonstrating their efficacy in reducing systemic inflammation and disease activity.

Further research is warranted to establish optimal dosing, duration of therapy, and long-term safety profiles of JAK inhibitors in this patient population. These preliminary results are encouraging and suggest a new avenue for treating AOSD.

**Key learning points:**

• **Efficacy of JAK inhibitors**: JAK inhibitors, particularly tofacitinib and baricitinib, demonstrated substantial clinical improvement in patients with refractory AOSD.

• **Steroid reduction**: All three patients were able to reduce or discontinue steroid use, highlighting the potential of JAK inhibitors to minimise long-term steroid dependency.

• **Management of refractory AOSD**: JAK inhibitors provided effective disease control where conventional therapies such as methotrexate, azathioprine, and biologics like tocilizumab and anakinra were inadequate or resulted in adverse effects.

• **Mechanism of action**: The efficacy of JAK inhibitors may be attributed to their ability to block pro-inflammatory cytokine pathways, particularly IL-6 and IFN, which are crucial in AOSD pathogenesis.

• **Safety profile**: The treatment was well-tolerated, with significant symptomatic relief and no severe adverse events reported during the study period.

• **Need for further research**: There is a need for further prospective studies to determine the optimal dosing, duration of therapy, and long-term safety profiles of JAK inhibitors in AOSD patients.

• **Personalised treatment approach**: The study supports a personalised treatment approach, considering JAK inhibitors for patients with difficult-to-treat AOSD who do not respond adequately to conventional therapies.

